# Anatomical Study of the Anastomosis Stations of Lateral Supramalleolar Artery Flap and Its Application in the Reconstruction of Distal Leg, Ankle, and Dorsum of Foot Defects

**DOI:** 10.1055/s-0045-1809152

**Published:** 2025-05-20

**Authors:** Thalavirithan Margabandu Balakrishnan, U. Raseedha Begum, Madhuvanti Sarada Bharathwaj, Madhumitha S., Pavithra Thangavel, M. Sugumar

**Affiliations:** 1 Department of Plastic Surgery, Coimbatore Medical College and Hospital, Coimbatore, Tamil Nadu, India; 2 Department of Plastic Surgery, Madras Medical college and Rajiv Gandhi Government General Hospital, Chennai, Tamil Nadu, India

**Keywords:** lateral supramalleolar artery flap, anastomotic stations, distal leg defects, ankle and dorsum of foot defects

## Abstract

**Introduction:**

A study was conducted to reveal the anatomy of the perforators and stations of anastomosis of the lateral supramalleolar artery (LSMA) axis and to analyze the outcome of various avatars of the flap based on the LSMA axis.

**Materials and Methods:**

Ten cadaveric dissections were performed to study the anatomy of the LSMA axis. Twenty patients with distal third leg, ankle, and dorsum of foot defects underwent reconstruction using the various avatars of the LSMA axis flaps. At the end of the follow-up, they were all assessed using the Institutional Functional and Aesthetic Outcome Assessment scoring system by two independent observers.

**Results:**

The cadaveric study showed consistent anatomical details of superficial peroneal nerve artery-LSMA-descending branch of ramus perforans anastomotic axis and their perforators. In the clinical study, the average size of the primary defect was 63.6 cm ^2^ . The average follow-up period was 12.5 months. Three (15%) noncritical complications were reported in our study, which were all managed conservatively. Fifty-five percent ( *n* = 11) of patients had a good/excellent final computed institutional assessment score ( *p* = 0.025). The rest of the patients (45% [ *n* = 9]) had a fair outcome score.

**Conclusion:**

Reconstructing soft tissue defects in the distal third of leg, ankle, and foot presents a considerable challenge. The LSMA flap and its various avatars may be a useful addendum in the reconstructive armamentarium for coverage of distal third leg, ankle, and dorsum foot defects.

## Introduction

**Fig. 1:**
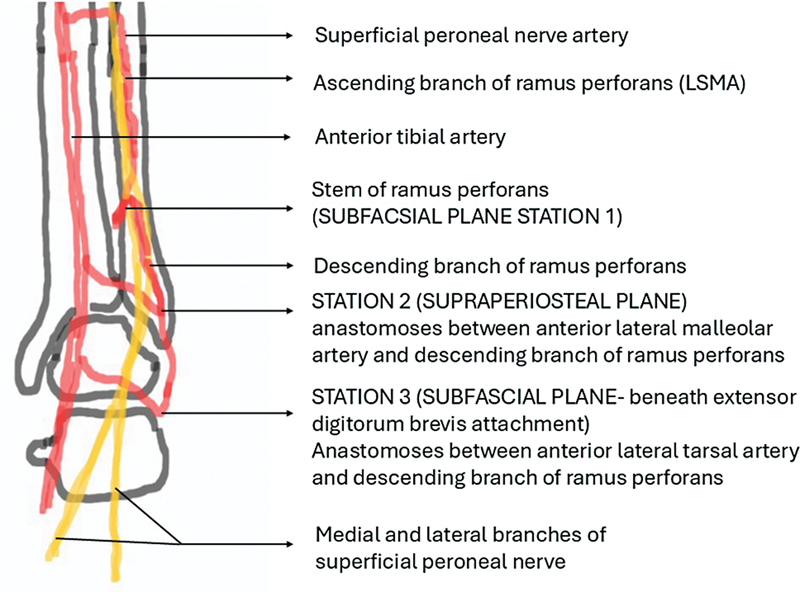
Schematic diagram showing station one, two, and three anastomoses for the lateral supramalleolar artery (LSMA) flap.

**Fig. 2:**
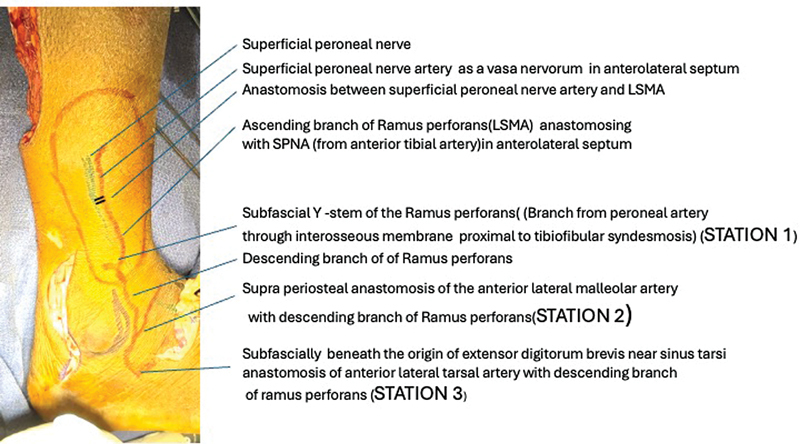
Surface marking of the various vascular stations and axis along the superficial peroneal nerve artery (SPNA)-lateral supramalleolar artery (LSMA)-descending branch of ramus perforans (DBRP) axis.

In the past decade, there has been a significant rise in the adoption of microvascular free tissue transfer to address soft tissue defects in the distal third leg region. Despite the array of available techniques today, considering the steep learning curve associated with microsurgery and the risk of free tissue transfer failure, employing local flaps remains crucial in the arsenal of plastic surgery. Among these options, the lateral supramalleolar artery (LSMA) flap stands out as a dependable choice for effectively addressing defects in the ankle, foot, and distal leg regions. The LSMA is the name given for the ascending branch of the ramus perforans artery, which in turn is a branch of the peroneal artery. 1 The LSMA flap was initially described by Masquelet et al in 1988 as a pedicled fasciocutaneous flap. 2 In 1991, Valenti et al modified the flap with a skin island and fasciocutaneous pedicle. 3 4 Later, in 2010, Lee and Chung described the reverse adipofascial modification of the flap, which helped to increase the distal reach of the flap. 5 There have been various modifications of this flap in the recent decade. The LSMA flap contains the superficial peroneal nerve (SPN; especially the lateral branch) making it a neuro-fasciocutaneous flap, and the vasa nervorum plexus in its substance, derived from the SPN artery (SPNA)—a branch from the anterior tibial artery. This vascular axis is reinforced by the ramus perforans (a branch of the peroneal artery) and its ascending and descending branches at station one. The vascular axis is further extended along the descending branch of ramus perforans (DBRP) to station two at the anterior aspect of lateral malleolus, where it is reinforced by the anterior lateral malleolar artery (a branch of the anterior tibial artery). It can even be extended to station three at the lateral end of the sinus tarsi, where it is reinforced by the anterior lateral tarsal artery (a branch from the dorsalis pedis artery) ( Fig. 1 ). The LSMA flap can be based on any of these stations. 4 The classical LSMA flap is a septofascioneurocutaneous flap, which includes the SPN at the middle third and lower third junction of the leg in the anterolateral septum. It is dissected up to and based on the subfascial Y stem of the ramus perforans artery, which forms station one ( Figs. 1 and 2 ). As the dissection proceeds distally and subperiosteally, after dividing the stem of the ramus perforans, it can be based on station two anastomosis ( Figs. 1 and 2 ). If the dissection continues in the subfascial plane, toward the sinus tarsi (station three anastomosis) ( Figs. 1 and 2 ), the length arc of rotation can be increased further to reach even the dorsum of the toes. The skin component of the flap extends from the junction of the middle and lower third of the leg proximally, which can be safely extended up to the midpoint of the leg, if there is a recruitment of sizable LSMA. 1 It can be safely extended distally up to station three at sinus tarsi. However, it should not go proximally to the middle third of the leg or extend beyond the tibial anterior border medially and 2 cm posterior to the fibula. 1 To this date, there is no literature available on the course, size, and distribution of perforators from the SPNA-LSMA-DBRP axis. This study encompasses the cadaveric dissection showing the various stations of anastomoses and individual perforators arising from these stations. We used the anatomical knowledge of the individual perforators from these stations/segments to raise different avatars of the LSMA flap including perforator propeller flaps from the individual perforators and assessed their use in providing soft tissue cover over the distal third leg, foot, and ankle defects.

## Materials and Methods

### Cadaveric Study

**Fig. 3:**
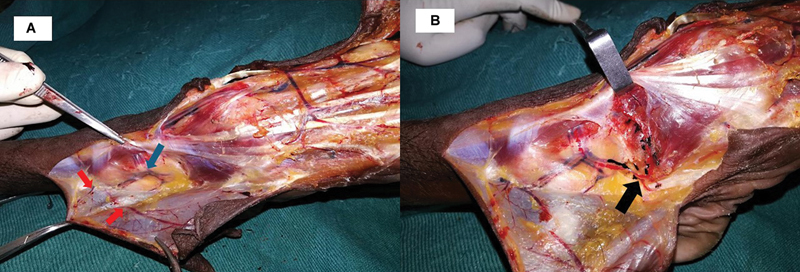
( **A** ) Cadaver dissection specimen showing two perforators (red arrows) from station one in relation to superficial peroneal nerve. Blue arrow showing perforator from the descending branch of ramus perforans just before station two anastomosis. ( **B** ) After reflecting the attachment of extensor digitorum brevis, the musculocutaneous perforator was shown (black arrow).

A preliminary anatomical study was conducted from March 2018 to March 2020 to study the course of ramus perforans, its ascending and descending branches, their stations of anastomoses around the ankle, and the perforator distribution in each segment. Five preserved and five fresh injected, adult cadaver specimens were examined by dissection. Cadaver specimens with injury in the LSMA territory were excluded from the study. A long incision was made from the midpoint of the leg to the dorsum of the foot extending from the anterior compartment close to the shin into the first web space. As the skin flaps were raised in the subfascial plane, perforators arising from each segment were noted ( Fig. 3A and B ). Their site, size, and distribution were recorded. Calipers were used for measuring the dimension of perforators at their origin ( Table 1 ).

**Table 1:** Perforators from individual stations—cadaveric dissection study

Perforasomes	Average number of perforators	Median location of perforator with respect to tip of lateral malleolus	Average size of perforators (mm)
Perforators from ramus perforans at station one (neurocutaneous and fasciocutaneous perforators)	1.7	4.75 cm cranial	1.2
Descending branch segment in front of lateral malleolus at station two (fasciocutaneous perforators)	1.6	1.25 cm cranial	1.2
Descending branch of ramus perforans segment up to sinus tarsi at station three (fasciocutaneous and musculocutaneous perforators)	1.5	2.2 cm caudal	1.1

### Clinical Study

From March 2022 to March 2024, 20 patients (13 males and 7 females) underwent LSMA flap cover for soft tissue defects over the distal leg, foot, and ankle region. Among them, 18 were posttraumatic and 2 were postinfective soft tissue defects. Patients who had injury in the LSMA territory were excluded from the study. The patients who had reconstruction defects with the LSMA flap following revascularization procedures were also excluded from the study. The permission for carrying out the cadaveric and clinical study was granted by the Institutional Ethics and Scientific Credential Committee (RS/3/2018).

#### Preoperative Preparation

**Fig. 4:**
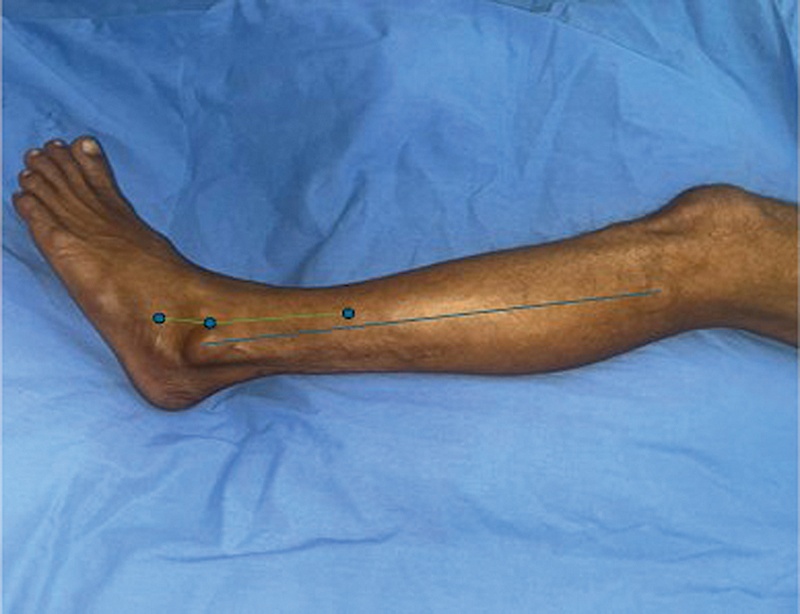
Surface marking for the vascular axis of lateral supramalleolar artery (LSMA) flap (red, axis of the LSMA flap; blue, axis of fibula).

The perforators were marked along the vascular axis surface marking of the flap using a handheld Doppler ( Figs. 2 and 4 ). This was done by marking the fibula from the midpoint of the lateral malleolus to the head of the fibula. A point was marked 1 cm anterior to the midpoint of the marked fibula axis and another at the extensor digitorum brevis (EDB) prominence (1.5 cm anterior and inferior to the tip of the lateral malleolus) and the two points were joined. This line should pass anterior to the anterior aspect of the lateral malleolus ( Fig. 4 ). All the patients were subjected to an anesthetic evaluation and underwent the procedure under spinal anesthesia. Patients were placed in supine position with the hip flexed and internally rotated and the knee flexed (one patient who underwent tendoachilles reconstruction in the same sitting was placed in the exaggerated lateral position for flap cover).

#### Surgical Technique

##### Classical Pedicled Flaps

The defects were debrided thoroughly, and the resultant defect was measured. Planning in reverse was done, and the skin paddle was marked centering the preoperative flap axis marking ( Fig. 3 ). Flaps were raised as peninsular or islanded based on the requirement, under tourniquet control. The SPN with SPN vessels were identified and ligated at the cranial end of the exploratory incision in the anterolateral septum. Dissection was then continued into the anterior and lateral compartments along the anterolateral septum subfascially, after retracting the peroneal muscles posteriorly-laterally and peroneus tertius anteriorly-medially, to identify the ascending branch of the ramus perforans up to its Y stem. For flaps based on the station one anastomosis, dissection was done up to 5 cm above the tip of the lateral malleolus. For flaps based on the station two anastomosis, dissection was done subperiosteally up to the tip of the lateral malleolus, protecting the subfascial anastomotic site. For flaps based on station three, subfascial dissection was extended up to the sinus tarsi. For the islanded flaps (usually in the tear drop configuration extending up to the pedicle base), the pedicle dissection was done in two planes—a deeper subperiosteal/subfascial dissection up to the anastomotic sites and superficial subdermal plane maintaining all the vascular plexus along the pedicle axis.

##### Perforator Propeller Flaps

The perforators were marked preoperatively using a handheld Doppler along the vascular axis that is described previously. A nondelineating incision was made in the lateral aspect of the skin paddle to identify the perforators along the vascular axis from various stations. Once the single best perforator was identified, the incision was completed on all sides and a subfascial/periperforator dissection was done, carefully preserving even the SPNA and SPN. In cases where a suitable perforator could not be identified, the flap was raised as a pedicled peninsular or islanded flap. If more than one perforator was located, the single best perforator was identified by microvascular clamping and assessing the perfusion at the business end of the flap (with a soft intestinal clamp applied at the undermined nondelineated area). After the single best perforator was identified, the other pedicles were ligated to facilitate the propeller movement of the flap. Flap inset was given incorporating interpolation primary movement (clockwise or anticlockwise, whichever permitted a more gracious turn of the pedicle without acute kinking or twisting) after ensuring good perfusion at the business end of the flap. The secondary donor defect was resurfaced with split-thickness skin graft harvested from the thigh. Sterile dressing was done with the below-knee posterior slab with ankle offloading to prevent compression on the pedicle. Monitoring the flap was done through a window dressing, and dressings were changed regularly.

#### Follow-Up

Patients were followed up weekly till removal of sutures, and then monthly. At the end of the follow-up, they were assessed by an objective Institutional Functional and Aesthetic Outcome Assessment score ( Table 2 ) by two independent observers. The final score was computed for each patient and recorded.

**Table 2:** Objective institutional, functional, and aesthetic outcome assessment score for foot reconstruction

Functional and aesthetic outcome		Score
Passive range of motion (ROM) in the subtalar joints (inversion and eversion) compared with uninjured limb at the end of follow-up	0 (zero) ROM	1
	< 5 degree (painful)	2
	5–10 degree (painful)	3
	Up to15 degree (full ROM, pain-free)	4
Passive ROM—plantar flexion and dorsiflexion at the talocrural joint compared with uninjured limb at the end of follow-up	0 (zero) ROM	1
	< 15 degree (painful)	2
	15–25 degree (painful)	3
	30–45 degree (pain-free)	4
Gait	Painful equinus/calcaneal gait with support	1
	Walking on reconstructed foot with occasional limping	2
	Normal swing and stance phase of the gait	3
Contour highlights of malleoli and heel region and shoeability	Nondiscernible and needs special shoes	1
	Moderately discernible and needs counter modified shoes	2
	Fairly discernible and using the normal shoes with difficulty	3
	Excellent/good definition and using normal street shoes regularly	4

Note: Poor outcome score: 4–6; fair outcome score: 7–10; good/excellent outcome score: 11–15.

#### Illustration of Cases

##### Case 1

**Fig. 5:**
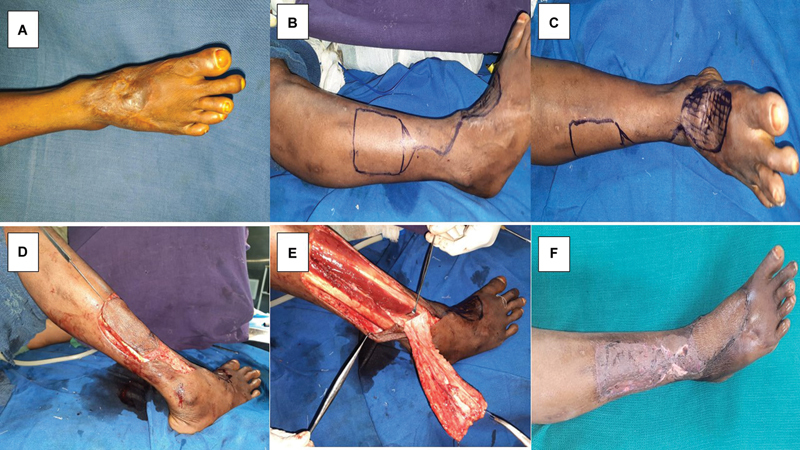
( **A** ) Preoperative picture of case 1, ( **B** and **C** ) islanded pedicled lateral supramalleolar artery (LSMA) flap marking, ( **D** and **E** ) raising of islanded flap in progress, and ( **F** ) late postoperative photograph.

A 30-year-old female patient presented with an unstable scar over the dorsum right foot and ankle following a scald burn ( Fig. 5A ). Scar excision was done. Islanded LSMA flap was marked ( Fig. 5B and C ). An islanded flap was raised based on station one and interpolated to the post-excisional defect ( Fig. 5D and E ). Flap settled well without any complications. The patient was followed up for 26 months ( Fig. 5F ). She regained a good range of motion in the ankle joint. She had an institutional assessment score of 8 at the end of the follow-up ( Table 3 ).

**Table 3:** Patient data

Case no.	Age	Sex	Etiology of defect	Site of defect	Flap dimensions (cm)	Vascular station	Configuration of flap	Follow-up period (mo)	Complications	Institutional outcome assessment score
1.	30	F	Posttraumatic/unstable scar	Ankle + dorsum of foot	12 × 6	1	Pedicled islanded	26	Nil	8 (fair)
2.	49	M	Postinfective	Tendoachilles region	10 × 6	2	Pedicled peninsular	8	Nil	10 (fair)
3.	35	M	Posttraumatic	Distal medial border of foot	19 × 5	3	Pedicled islanded	9	Nil	14 (good/excellent)
4.	25	M	Posttraumatic	Anterior ankle + distal leg	10 × 8	1	Perforator propeller flap	26	Nil	15 (good/excellent)
5.	22	M	Posttraumatic	Dorsum of foot	9 × 7	2	Perforator propeller	12	Nil	8 (fair)
6.	45	M	Posttraumatic	Anterior ankle and dorsum of foot	9 × 4	3	Perforator propeller flap	12	Nil	9 (fair)
7.	45	F	Posttraumatic	Distal foot dorsum	10 × 7	3	Pedicled islanded	16	Wound infection	11 (good/excellent)
8.	58	M	Posttraumatic	Anterior distal leg	8 × 6	1	Pedicled peninsular	8	Nil	12 (good/excellent)
9.	44	M	Posttraumatic	Ankle + lower leg	12 × 8	1	Perforator propeller	18	Nil	9 (fair)
10.	35	F	Posttraumatic	Ankle	10 × 8	1	Pedicled peninsular	9	Nil	10 (fair)
11.	29	M	Posttraumatic	Dorsum of foot	9 × 6	2	Pedicled islanded	13	Nil	12 (good/excellent)
12.	36	F	Posttraumatic	Dorsum of foot	5 × 8	2	Pedicled peninsular	7	Nil	11 (good/excellent)
13.	58	M	Posttraumatic	Distal leg	11 × 7	1	Pedicled islanded	14	Wound dehiscence	10 (fair)
14.	35	M	Posttraumatic	Distal foot	8 × 5	3	Pedicled islanded	7	Nil	11 (good/excellent)
15.	58	M	Posttraumatic	Lower leg	12 × 7	1	Perforator propeller	8	Nil	13 (good/excellent)
16.	41	F	Posttraumatic	Ankle	11 × 6	3	Perforator propeller	8	Nil	9 (fair)
17.	44	M	Postinfective	Dorsum of foot	15 × 7	2	Perforator propeller	7	Superficial epidermolysis	11 (good/excellent)
18.	26	M	Posttraumatic	Ankle	8 × 4	2	Perforator propeller	11	Nil	8 (fair)
19.	37	M	Posttraumatic	Distal foot dorsum	7 × 5	3	Pedicled peninsular	16	Nil	14 (good/excellent)
20.	31	F	Posttraumatic	Ankle	8 × 5	1	Perforator propeller	10	Nil	12 (good/excellent)

Abbreviations: F, female; M, male.

##### Case 2

**Fig. 6:**
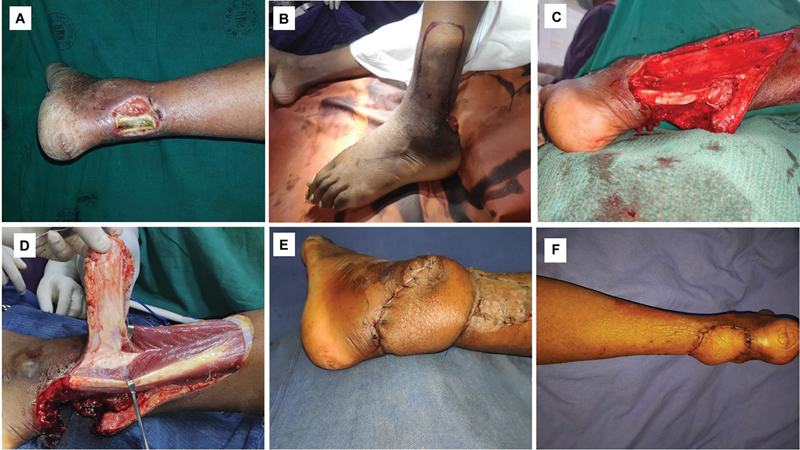
( **A** ) Preoperative photograph of case 2 showing sloughed out exposed tendoachilles, ( **B** ) lateral supramalleolar artery (LSMA) pedicled peninsular flap marking, ( **C** ) reconstruction of tendon gap with inert iliotibial fascial graft, ( **D** and **E** ) pedicled peninsular flap elevation in progress, and ( **F** ) late postoperative picture.

A 49-year-old male patient presented with post infective defect over the tendoachilles region exposing the devitalized tendoachilles ( Fig. 6A ). After thorough excisional and lavage debridement, converting the chronic infected wound to acute clean wound, the LSMA flap was planned ( Fig. 6B ). The tendoachilles tendon gap was reconstructed with an inert iliotibial tract fascial graft ( Fig. 6C ). A pedicled peninsular LSMA flap based on station two was used for the reconstruction of the defect ( Fig. 6D and E ). The flap settled well. He was followed up for 8 months ( Fig. 6F ). At the end of the follow-up, he was able to stand up on the toes of the reconstructed limb alone without support (suggestive of integrity of the triceps surae musculotendinous unit). He had an institutional assessment score of 10 at the end of the follow-up ( Table 3 ).

##### Case 3

**Fig. 7:**
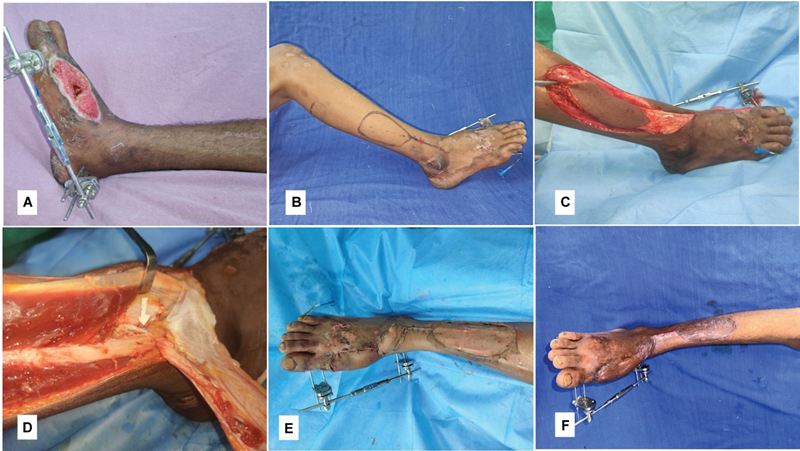
( **A** ) Preoperative picture of case 3, ( **B** ) pedicled islanded lateral supramalleolar artery (LSMA) flap marking, ( **C** and **D** ) flap elevation beyond station two exposing the anterior aspect of the lateral malleolus, ( **E** ) final inset, and ( **F** ) late postoperative picture.

A 35-year-old male patient with a posttraumatic soft tissue defect over the tibial border of the distal foot following fracture dislocation tarsometatarsal joint. External fixator was placed for stabilization ( Fig. 7A ). After the debridement, and planning in reverse, a tear-shaped pedicled islanded LSMA flap was marked ( Fig. 7B ). The flap was based on station three ( Fig. 7C and D ). The donor site defect and proximal pedicle were resurfaced with split-thickness skin graft ( Fig. 7E ). The flap settled well with no complications. The patient was followed up for 9 months ( Fig. 7F ). The final computed institutional assessment score was 14 at the end of the follow-up ( Table 3 ).

##### Case 4

**Fig. 8:**
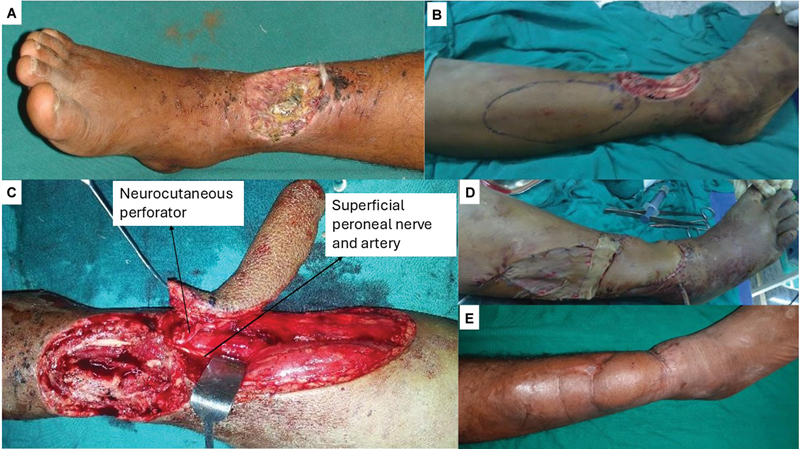
( **A** ) Preoperative picture of case 4, ( **B** ) preoperative marking of perforator propeller flap, ( **C** ) perforator propeller flap raised on single best neurocutaneous perforator preserving the superficial peroneal nerve (SPN) and superficial peroneal nerve artery (SPNA), ( **D** ) after propeller movement and inset, and ( **E** ) late postoperative picture.

A 25-year-old male presented to us with a posttraumatic wound over the anterior aspect of the ankle and distal leg segment ( Fig. 8A ). The perforator was located just cranial to the wound using a 10-MHz Doppler and a perforator propeller flap was marked ( Fig. 8B ). The flap was raised on a single best neurocutaneous perforator, preserving the SPN and SPNA ( Fig. 8C ). This perforator was found very close to the Y stem of ramus perforans at station one ( Fig. 8C ). The secondary defect was resurfaced with the split-thickness skin graft after 180-degree primary propeller movement and inset ( Fig. 8D ). The flap settled well and he was followed for 26 months ( Fig. 8E ). At the end of the follow-up, the computed institutional assessment score was 15 ( Table 3 ).

##### Case 5

**Fig. 9:**
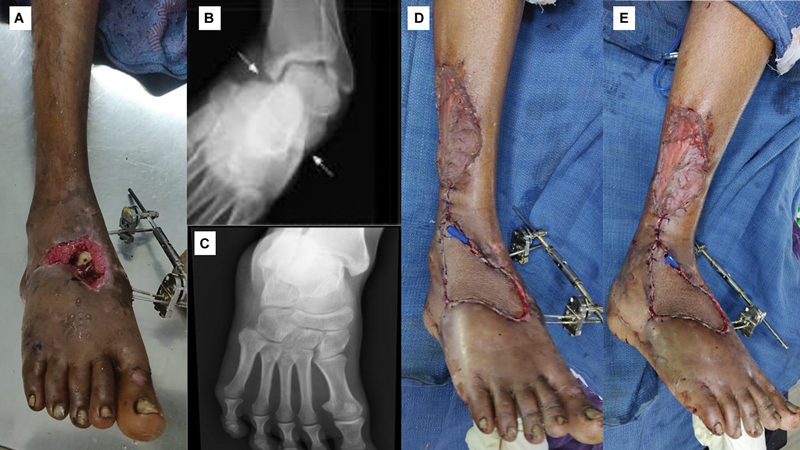
( **A** ) Preoperative picture of case 5, ( **B** and **C** ) X-ray showing subtalar subluxation and reduction, and ( **D** and **E** ) postoperative results of the cover of soft tissue defect and stabilization of the dorsum with external fixator.

A 22-year-old male sustained a right subtalar subluxation ( Fig. 9B and C ) following a road traffic accident, with a raw area over the dorsum of the foot exposing the medial and intermediate cuneiform bones, second tarsometatarsal joint, and base of the third metatarsal bone ( Fig. 9A ). After stabilizing the foot with an external fixator ( Fig. 9A ), a perforator propeller flap based on the station two perforator was performed ( Fig. 9D and E ). He developed severe stiffness at the talocrural and subtalar joints. He could walk with a short stride gait at the end of a 12-month follow-up period. At the end of the follow-up, he had a final computed score of 8 ( Table 3 ).

##### Case 6

**Fig. 10:**
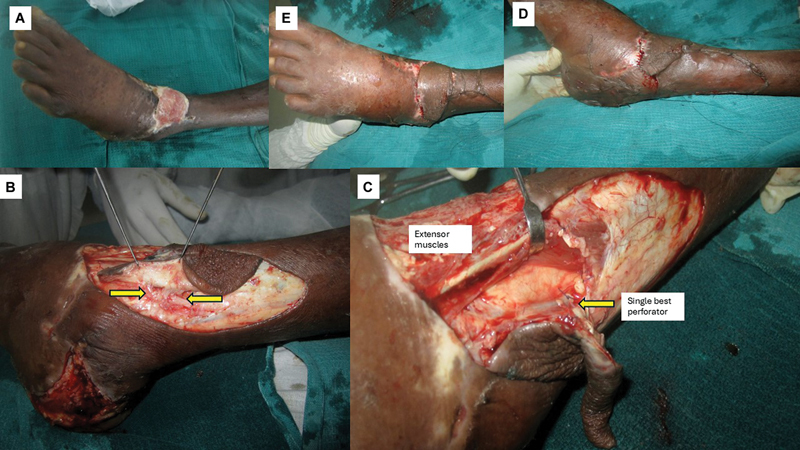
( **A** ) Preoperative picture of case 6, ( **B** ) the nondelineating dissection showing two perforators in the vicinity of station three (yellow arrows), ( **C** ) flap raised on single best musculocutaneous perforator (yellow arrow), and ( **D** and **E** ) final inset.

A 45-year-old male with controlled systemic hypertension presented with a posttraumatic wound exposing the extensor tendons at the ankle extending on to the dorsum of the foot ( Fig. 10A ). A perforator propeller flap was planned based on the perforators along the descending branch of the ramus perforans over the knuckle of the EDB muscle. A nondelineating exploratory incision and subfascial/subcutaneous dissection showed two perforators in the vicinity of station three anastomosis ( Fig. 10B ). The flap was raised on the single best musculocutaneous perforator arising from the EDB muscle ( Fig. 10C ). It was propelled into the defect and inset was given ( Fig. 10D and E ). The patient was followed up for 12 months and the flap settled well with no complications. His institutional assessment score at the end of the follow-up period was 9 ( Table 3 ).

## Results

### Cadaveric Study

We found that the station one Y stem of ramus perforans was consistently present in all cadaveric specimens at an average distance of 4.25 cm cranial to the tip of the lateral malleolus. The anastomosis between SPNA and ascending branch of ramus perforans (LSMA) along the SPN stem and its lateral branch in the station one segment gave rise to an average of 1.7 perforators ( Fig. 2A ) ( Table 1 ). The station two anastomotic source vessels (anterior lateral malleolar artery and descending branch of the ramus perforans) were found in the subfascial plane on the periosteum covering the anterior aspect of the lateral malleolus. Later envisaged, the base of the pedicled (peninsular/islanded) flaps on station two anastomosis must be raised distally in the subperiosteal plane protecting the anastomotic source vessels. However, the pedicled (peninsular/islanded) flaps based on station one should be raised as septofasciocutaneous flaps, including the SPNA-LSMA vessel axis at the base of the anterolateral septum (because the SPN initially runs at the middle third-lower third leg junction in the base of the anterolateral septum). The station three anastomosis was present at the sinus tarsi in all specimens beneath the proximal attachment of the EDB muscle. Therefore, for the pedicled (peninsular/islanded) flaps based on station three, after retracting the extrinsic extensor (peroneus tertius and extensor digitorum longus) muscles medially, the subfascial dissection must be continued over the capsulo-ligamental complex on the anterolateral aspect of the talocrural joint ( Fig. 2B ). The longest segment was found to be the station one segment and the shortest was found to be the station three segment. The average length of the station one segment was 10.2 cm. The average length of the station two segment was found to be 5.5 cm, and the average length of the station three segment was 2.9 cm. The neurocutaneous perforators of station one arises from the anastomotic chain between the SPNA and LSMA. The robust perforators were located closer toward the caudal end of station one. In all the stations, perforator flaps could be dissected with the preservation of SPN and the source vessels. Table 1 shows further anatomical details of the perforators.

### Clinical Study

Twenty patients underwent pedicled LSMA flap—islanded ( *n* = 6), peninsular ( *n* = 5), and perforator propeller flap ( *n* = 9) for soft tissue defects over the distal leg, ankle, and foot. Age of the patients ranged from 22 to 58 years. Six patients were female and 14 were male. Associated chronic comorbidities were systemic hypertension ( *n* = 3) (15%), diabetes mellitus ( *n* = 5) (25%), and alcoholic chronic liver disease ( *n* = 1) (5%). Two (10%) patients had stopped smoking 3 weeks prior to the surgery. Among them, 18 were posttraumatic (90%) and 2 were postinfective soft tissue defects (10%). Five patients underwent pedicled flaps based on station one ( Table 3 ). Three patients underwent pedicled flaps based on station two and the rest ( *n* = 3) underwent pedicled flaps based on station three ( Table 3 ). Among the perforator propeller LSMA flaps, four were based on station one segment, three were based on station two segment, and two were based on station three segment. The average size of the defect in this study was 63.6 cm ^2^ (ranging from 8 × 4 cm to 15 × 7 cm) ( Table 3 ). The average follow-up period in our study was 12.5 months. Anesthesia in the distribution of the SPN (which was an expected outcome in all pedicled peninsular/islanded flaps) was a significant problem in only two (10%) patients. Apart from this, three (15%) noncritical complications ( Table 3 ) were reported in our study, which were all managed conservatively. The institutional Objective Functional and Aesthetic Outcome Assessment Score for Foot Reconstruction ( Table 2 ) was evaluated using Cronbach's alpha to determine both internal consistency and interrater reliability and was found to be 0.75. The Pearson's correlation coefficient was 0.8 when calculated for all four arms of the scoring system for the evaluation of strong positive relationship between two observer findings. Fifty-five percent ( *n* = 11) of patients had a good/excellent final computed institutional assessment score ( *p* = 0.025 as assessed by Student's *t* -test). The rest of the patients (45% [ *n* = 9]) had a fair outcome score. Our analysis of the outcome showed that those who had a fair score had sustained high-energy skeletal injures contributing to the poorer outcome. However, there was a 100% survival of all the flaps in our study ( Table 3 ).

## Discussion

The LSMA flap is based on nonaxial source vessel's anastomotic chain that includes craniocaudally the SPNA, LSMA, and DBRP. In addition, the perforator propeller flaps based on the perforator from the three stations/segments are endowed with the supranormal homogenous blood supply along with the preservation of SPN and the source vessel anastomotic chain at the donor site. 6 7 8 9 Our study encompassed pedicled islanded, pedicled peninsular, and perforator propeller flaps based on the SPNA-LSMA-DBRP anastomotic chain and, therefore, no axial vessels were sacrificed. All flaps from this anastomotic axis were local flaps and hence facilitated a good color, thickness, and texture match reconstruction. The reconstruction of the distal third of leg, ankle, and dorsum of foot regions envisage good reconstitution of contour highlights with reestablishment of preinjury range of mobility in the regional joints. 10 Wound coverage for distal third leg defects has been a challenging problem over the last few decades before the advent of microsurgery. 5 This is largely due to the anatomy of this region. Muscles of the leg become tendinous around this region, making it mandatory for flap cover. Fractures are common in this region following trauma due to the sparse and thin, soft tissue cover and protean subcutaneous bony and tendon prominences. Adept reconstruction can be achieved only by thin, pliable local flaps with minimal scarring.

The LSMA flap has extensive versatility to satisfy all the above requirements. Our clinical and cadaveric study is the first of its kind in establishing the anatomical basis, surface marking, techniques of safe dissection, and designing the latest avatar from the LSMA perforasome—the LSMA perforator propeller flaps. An extensive study of the vascular system of this flap performed by Rong et al 11 described the fasciocutaneous and direct cutaneous perforators from these stations. However, our study establishes more detailed anatomy of the perforators in the three segments/stations and elaborates safe techniques for raising the various avatars of the LSMA flap. Local flap options for distal third of leg, ankle, and foot defects are lateral supramalleolar flaps, reverse sural artery flap, peroneus brevis muscle flap, and crossed leg flaps. 12 13 14 The crossed leg flap is not commonly used now, due to the difficulty in maintaining position and poor patient compliance. 15 The LSMA flaps were chosen over the reverse sural artery flap 12 13 14 for the following reasons: (1) the lateral supramalleolar flap can be used for mixed flow and retrograde flow patterns due to anatomical characteristics of the blood supply, and also the incidence of venous congestion is less; (2) the learning curve for the procedure is short; and (3) the procedure can be done in supine position easily. The LSMA flap and its various avatars stand out among these choices because of its versatility, which facilitates shoeability, minimal donor site contour deformity, and effectively promotes healing of underlying skeletal and tendon injuries as shown in our study. Various authors 16 17 18 19 have described the use of pedicled LSMA flaps only, but in our study, we have analyzed the results of various forms of flaps from the SPNA-LSMA-DBRP axis perforasomes. In the last decade, microsurgical reconstruction has superseded in use and benefits of local flaps. 20 Nevertheless, local flaps, especially the perforator propeller flaps (microvascular flaps without microvascular anastomoses), have carved a niche in the armamentarium of plastic surgeons as they are less technically demanding and less labor intensive. However, the disadvantages of the LSMA flap described in the literature were that it has a limited skin component, reduced bulk, and increased incidence of venous congestion. 16 17 18 19 By understanding the anatomy of the anastomotic chain between SPNA-LSMA-DBRP perforasomes and ensuring safe methods of elevation described in our study, one can minimize these disadvantages and limitations. In our study, the lateral supramalleolar flap was used in the soft tissue coverage of a wide range of defects in the distal third of leg, ankle, and foot with good results. The LSMA flap showed a wide arc of rotation with the distal most part reaching up to just distal to the metatarsophalangeal joint of the first toe when based on anastomosis from station three. The distal and medial reach of the flap was increased by islanding the flap. However, we found that large, islanded flaps are more prone to venous congestion due to fragility of the pedicle. This can be reduced by converting larger flaps based on retrograde flow to peninsular flaps. As per our study, we would like to recommend the choice for flaps as follows—station three-based flaps for small to medium distal foot defects and dorsum of foot defects; station two-based flaps for dorsum of foot defects and larger, distal periankle defects; and station one-based flaps for small to medium proximal ankle defects and lower leg defects.

The strength of our study is establishing the anatomy of perforators and subsequently formatting the designing and construction of the flaps based on the knowledge gleaned from the anatomical study. The outcome was also assessed by our statistically validated objective Institutional Functional and Aesthetic Outcome Assessment score ( Table 2 ) by two independent observers. The Finnish version of the FAOS (the Foot and Ankle Outcome Score) system 21 is a popular one but assesses only patient-reported outcomes. It has no objective component. It was also designed for the skeletal restoration and not for the soft tissue reconstruction in the foot and ankle region. But our scoring system is specific for the later. The limitation of our study is the smaller sample size. It may need a larger sized study to establish further the versatility of the various avatars of the LSMA flap.

## Conclusion

Reconstructing soft tissue defects in the distal third of leg, ankle, and foot presents a considerable challenge. The cadaveric study is a flip to understanding the anatomy of perforators along the SPNA-LSMA-DBRP axis. The LSMA flap and its various avatars may be a useful addendum in the reconstructive armamentarium for coverage of distal third leg, ankle, and foot defects.
